# Outcomes of Descemet Membrane Endothelial Keratoplasty for Vitrectomized Eyes with Sutured Posterior Chamber Intraocular Lens

**DOI:** 10.1155/2018/3127126

**Published:** 2018-04-11

**Authors:** Norihiro Yamada, Takahiko Hayashi, Kentaro Yuda, Toshiki Shimizu, Itaru Oyakawa, Hidenori Takahashi, Naoko Kato

**Affiliations:** ^1^Department of Ophthalmology, Saitama Medical University Hospital, Saitama, Japan; ^2^Department of Ophthalmology, Yokohama City University Hospital, Yokohama, Japan; ^3^Department of Ophthalmology, Yokohama Minami Kyosai Hospital, Yokohama, Japan; ^4^Kikuna Yuda Eye Clinic, Yokohama, Japan; ^5^Department of Ophthalmology, Jichi Medical University, Tochigi, Japan; ^6^Department of Ophthalmology, Heart Life Hospital, Okinawa, Japan

## Abstract

**Purpose:**

To evaluate the clinical outcomes of Descemet membrane endothelial keratoplasty (DMEK) for vitrectomized eyes that underwent pars plana vitrectomy (PPV) and transscleral-sutured intraocular lens (IOL) implantation.

**Methods:**

In this retrospective study, DMEK cases were reviewed from medical records and divided into two groups: the eyes after PPV and transscleral-sutured IOL implantation (vitrectomized group) and the eyes with in-the-bag IOL implantation (control group) prior to DMEK. The main outcome measures included time of graft unfolding during surgery and best spectacle-corrected visual acuity (BSCVA), central corneal thickness (CCT), and endothelial cell density (ECD) at 1, 3, and 6 months after the DMEK.

**Results:**

Twenty-three eyes (vitrectomized group, *n* = 8; control group, *n* = 15) in 23 patients were included in this study. The unfolding time was significantly longer in the vitrectomized group than in the control group (*P* < 0.001). Postoperative BSCVA was worse in the vitrectomized group (0.16 ± 0.15) than in the control group (−0.06 ± 0.06; *P* = 0.017). The improvement in BSCVA was negatively correlated with the patients' age and frequency of previous surgeries.

**Conclusions:**

Despite the longer graft unfolding time and limited visual recovery, DMEK should be applicable to vitrectomized eyes with transscleral-sutured IOL implantation.

## 1. Introduction

Descemet membrane endothelial keratoplasty (DMEK) is a new method of corneal endothelial keratoplasty introduced by Melles et al. [1], which allows rapid recovery of visual acuity and minimizes immunological rejection [1–5]. The surgical steps in DMEK include careful graft preparation, safe graft insertion into the anterior chamber, recognition of graft orientation, smooth graft unfolding, and successful graft attachment in the anterior chamber by air or gas tamponade [6–13].

The eyes that are most suitable for DMEK are thought to be pseudophakic bicameral eyes with normal anterior chamber depth. When DMEK is performed on an eye containing a sutured intraocular lens (IOL), the IOL should be properly centered within the lens capsule, providing an intact iris-IOL diaphragm. Although the indications of DMEK have been widely expanded to many endothelial disorders, the eyes with iris abnormalities or sutured IOL are thought to be unsuitable for DMEK [14–18], because the graft might be lost through the peripheral iris defect or the interspace between the iris and fixed IOL [18].

Vitrectomized eyes are further challenging because the absence of vitreous pressure during surgery requires unfolding and attaching the graft using air, which is difficult. Previous reports indicated that higher rates of postoperative graft dislocation were observed following DMEK for vitrectomized eyes and required rebubbling, resulting in a higher incidence of primary graft failure [15, 18]. However, some eyes develop bullous keratopathy that requires suturing of IOLs as well as pars plana vitrectomy (PPV) prior to endothelial keratoplasty.

The purpose of the present study was to determine the clinical outcomes, postoperative complications, and features of DMEK in the eyes that underwent previous PPV and transscleral-sutured IOLs.

## 2. Material and Methods

### 2.1. Participants

This was a retrospective study, and the study protocol was approved by the Institutional Review Board of Yokohama Minami Kyosai Hospital (approval number 29_03_05) and Saitama Medical University (approval number 17-032). The research followed the tenets of the Declaration of Helsinki. Patients with bullous keratopathy who underwent DMEK at the Yokohama Minami Kyosai Hospital and Saitama Medical University Hospital from January 2016 to December 2016 and who were followed up for more than 6 months were retrospectively analyzed. In this analysis, the eyes were classified into two groups based on the status of the IOL. The “vitrectomized” group consisted of patients with eyes that underwent PPV and transscleral-sutured IOL implantation before DMEK. The control group consisted of patients who had routine IOL implantation before DMEK. The eyes that underwent previous trabeculectomy or penetrating keratoplasty or had a history of birth injury or endotheliitis were excluded. Patients who did not agree with this study or could not be followed up were not included.

### 2.2. Surgical Procedure

#### 2.2.1. Donor Preparation

BBG 250® (BBG; Sigma-Aldrich, St. Louis, MO, USA) was dissolved in balanced saline solution (BSS® or BSS-plus®; Alcon, Osaka, Japan) to 0.1% (*w/v*). All grafts were peeled as described previously. BBG (0.1%, *w/v*) was used to stain the graft edges during peeling. A punch was gently placed on the endothelial surface to indent a circle 7.75, 8.0, or 8.25 mm in diameter. Subsequently, 1.0 and 1.5 mm- diameter dermatological biopsy punches (Kai Industries, Seki, Japan) were used to place asymmetric marks on the edges of the identified circles [19]. Donor grafts thus marked were stained with 0.1% (*w/v*) BBG (1.0 mg/mL) for 1 min and stored in BSS prior to insertion, 30 min later [20]. Because unfolding time depends on donor age, we usually selected donors over 60 years old.

#### 2.2.2. Surgical Techniques

All surgeries were performed under local anesthesia. After establishing retrobulbar anesthesia and Nadbath facial nerve block, two paracenteses and a 2.8 mm upper corneal or corneoscleral incision were made for the recipient cornea. Peripheral iridotomy was performed at the 6 o'clock position to prevent a postoperative pupillary block. The donor membrane graft, stained with 0.1% (*w/v*) BBG (1.0 mg/mL), was placed into an IOL injector (model WJ-60M; Santen Pharmaceuticals, Osaka, Japan) and inserted into the anterior chamber.

The inserted graft was unfolded using a noncontact technique by shallowing the anterior chamber [13]. In the vitrectomized group, if shallowing the anterior chamber was difficult, a small amount of air was also injected between the host cornea and donor graft, and the rolled up donor graft was subsequently unfolded. After the graft was unfolded, additional air was added slowly underneath the graft from the center, and the rolled graft was gradually attached to the host cornea. The folded edges of the graft were additionally stretched using “bubble-bumping maneuver” [13]. In cases of severe bullous keratopathy, we used the chandelier illumination technique during DMEK surgery via the pars plana approach [21]. After the correct orientation was confirmed, the anterior chamber was filled with air to adhere the graft to the host cornea. Fifteen minutes later, the air was partially replaced with BSS. Finally, 0.4 mg of betamethasone (Rinderon®; Shionogi, Osaka, Japan) was subconjunctivally administered in 1.5% (*w/v*) levofloxacin eye drops (Cravit®; Santen Pharmaceuticals).

Postoperative medications included 1.5% (*w/v*) levofloxacin (Cravit) and 0.1% (*w/v*) betamethasone sodium phosphate (Sanbetasone®; Santen Pharmaceuticals) commencing at four times daily for 3 months and tapering thereafter.

#### 2.2.3. Postoperative Follow-Up and Examinations

In addition to the standard ophthalmic examination, the best spectacle-corrected visual acuity (BSCVA), corneal endothelial cell density (ECD), central corneal thickness (CCT), and graft adaptation were evaluated both preoperatively and for up to 6 months postoperatively in all eyes. BSCVA was measured as decimal visual acuity and was converted to a logarithm of the minimum angle of resolution (LogMAR) values. Graft adaptation was assessed with both slit-lamp microscopy and an anterior segment OCT (SS1000; Tomey, Nagoya, Japan). Corneal thickness was measured by corneal tomography (SS1000; Tomey). When progressive graft detachment occurred near the central area, a rebubbling procedure was performed as described previously [22]. Preoperative ECD values were retrieved from donor eye bank records, and postoperative ECD values were measured with the aid of a specular microscope (FA3509; Konan Medical, Nishinomiya, Japan). Spectral-domain optical coherence tomography (RS 3000; Nidek, Japan) was performed 1, 3, and 6 months after DMEK. When CME was diagnosed postoperatively, topical bromfenac (Bronuck®, Senju, Pharmaceutical Co., Osaka, Japan) and sub-Tenon injection of triamcinolone acetonide (MaQaid®; Wakamoto Pharmaceutical Co., Tokyo, Japan) were immediately applied.

#### 2.2.4. Graft Unfolding Time

The graft unfolding time was evaluated using surgical videos and compared between the two groups. The time from the first tap used to unfold the tissue to the start of air injection underneath the graft was measured and defined as the unfolding time.

#### 2.2.5. Statistical Analysis

The Wilcoxon test or paired *t*-test was used to compare values preoperatively and postoperatively, as appropriate. The Mann–Whitney *U* test or unpaired t-test was used to compare values between the two groups, as appropriate. Due to the distribution of unfolding times, they were compared using the two-sided Student's *t*-test after logarithmic transformation. The male/female and right/left ratios were compared using the chi-square test. To explore related factors, multiple regression analysis with stepwise variable selection (minimum Bayesian information criterion, increasing number of variables) was performed. All analyses were performed using JMP Pro Software version 11.2.0 (SAS Institute, Cary, NC, USA). A *P* value < 0.05 was considered statistically significant.

## 3. Results

### 3.1. Patients

Twenty-three eyes in 23 patients (8 men and 15 women) were considered eligible for the study. Eight eyes came from the vitrectomized group, and the other fifteen eyes came from the control group. Ages ranged from 52 to 82 years (mean, 73.8 years). Preoperative patient profiles of the two groups are summarized in Table 1.

In the present study, the frequency of previous surgeries prior to DMEK was 3.11 ± 0.78 in the vitrectomized group. In contrast, only phacoemulsification and simultaneous IOL implantation were performed in the control group. The comparison between the two groups was statistically significant (*P* < 0.001).

#### 3.1.1. Vitrectomized Group

Eight eyes had previously undergone PPV and transscleral-sutured IOL implantation (vitrectomized group). Five of the eight eyes showed an aphakic state derived from complicated cataract surgeries, one eye underwent phacoemulsification and aspiration for pseudoexfoliation syndrome, and two eyes underwent intracapsular cataract extraction. These latter three eyes revealed pseudoexfoliation corneal endotheliopathy. One of the three eyes underwent aspiration for congenital cataract during childhood, after which the patient used a hard contact lens for a long time. Another eye was implanted with an IOL that was subluxated, which required the extraction of the lens and the secondary implantation of a new lens.

In three eyes, bullous keratopathy was caused by vitreoretinal surgeries; two had undergone pars plana vitrectomy for rhegmatogenous retinal detachment with silicone oil injection, while one had pars plana vitrectomy with silicone oil injection for endophthalmitis after cataract surgery. Detailed patient profiles of the vitrectomized group are summarized in Table 2.

#### 3.1.2. Control Group

Fifteen eyes underwent routine cataract surgery with in-the-bag IOL implantation prior to DMEK (control group). Six eyes had Fuchs' corneal endothelial dystrophy, nine had iatrogenic bullous keratopathy, six underwent argon laser iridotomy, and three were subjected to phacoemulsification and IOL implantation.

### 3.2. Visual Acuity

**Figure 1:**
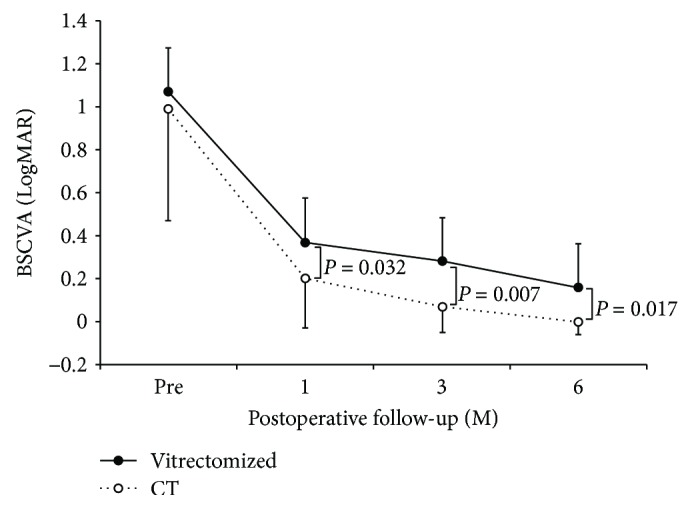
Changes in best spectacle-corrected visual acuity (BSCVA). Statistically significant improvement in BSCVA was seen in the vitrectomized group (*P* = 0.011 at 1 month, 0.005 at 3 months, and 0.003 at 6 months; Wilcoxon rank sum test). In the control group, a statistically significant improvement of BSCVA was seen at all observation points (*P* = 0.002 at 1 month, 0.001 at 3 months, and 0.001 at 6 months; Wilcoxon rank sum test). There was also a statistically significant difference in BSCVA between the two groups at all postoperative examinations (*P* = 0.795 preoperatively, 0.032 at 1 month, 0.007 at 3 months, and 0.017 at 6 months; Mann–Whitney *U* test). Vitrectomized: vitrectomized group; CI: confidence interval; CT: control group.

In the vitrectomized group, BSCVA improved from 1.15 ± 0.60 preoperatively to 0.37 ± 0.19 at 1 month, 0.28 ± 0.15 at 3 months, and 0.16 ± 0.15 at 6 months. In the control group, BSCVA improved from 0.98 ± 0.52 preoperatively to 0.20 ± 0.23 at 1 month, 0.07 ± 0.12 at 3 months, and −0.06 ± 0.06 at 6 months. A statistically significant improvement in BSCVA was obtained in the vitrectomized group at all observation points (*P* = 0.011 at 1 month, 0.005 at 3 months, and 0.003 at 6 months). A statistically significant improvement of BSCVA was also obtained in the control group at all observation points (*P* = 0.002 at 1 month, 0.001 at 3 months, and 0.001 at 6 months). The BSCVA in the control group was significantly better than that in the vitrectomized group at all the examination points (*P* = 0.795 preoperatively, 0.032 at 1 month, 0.007 at 3 months, and 0.017 at 6 months; Figure 1).

### 3.3. Central Corneal Thickness

In the vitrectomized group, the CCT decreased from 764.5 ± 62.7 *μ*m preoperatively to 529.5 ± 56.6 *μ*m at 1 month, 520.6 ± 51.6 at 3 months, and 513.3 ± 43.3 at 6 months. In the control group, the CCT decreased from 722.0 ± 88.8 *μ*m preoperatively to 555.9 ± 64.8 *μ*m at 1 month, 507.3 ± 53.7 at 3 months, and 513.8 ± 52.9 at 6 months. A statistically significant improvement in CCT was observed in each group at all examination points (*P* < 0.001, Wilcoxon rank sum test in both groups). There was no significant difference in CCT between the two groups at any examination point. The *P* values were 0.194 preoperatively, 0.136 at 1 month, 1.0 at 3 months, and 0.810 at 6 months.

### 3.4. Corneal Endothelial Cell Density

**Figure 2:**
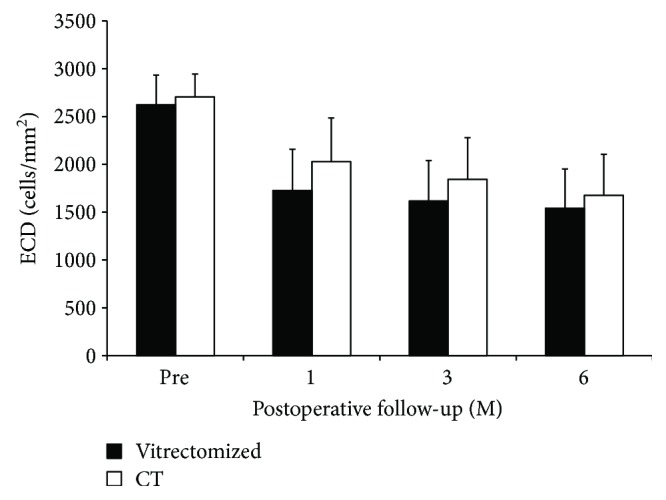
Changes in endothelial cell density (ECD). In the vitrectomized group, the donor corneal ECD decreased from 2629 ± 303 cells/mm^2^ preoperatively to 1548 ± 401 cells/mm^2^ at 6 months postoperatively (40.7 ± 11.2% less than the preoperative value of the donor graft). In the control group, the donor corneal ECD decreased from 2707 ± 238 cells/mm^2^ at preoperative point to 1679 ± 419 cells/mm^2^ at 6 months postoperatively (38.2 ± 18.6% less than the preoperative value of the donor graft). There was no significant difference in ECD between the two groups at any pre- and postoperative points (*P* value = 0.832 preoperatively, 0.136 at 1 month, 0.259 at 3 months, and 0.526 at 6 months; Mann–Whitney *U* test). Vitrectomized: vitrectomized group; CI; confidence interval; CT; control group.

In the vitrectomized group, the donor corneal ECD decreased from 2629 ± 303 cells/mm^2^ preoperatively to 1728 ± 429 cells/mm^2^ at 1 month, 1620 ± 414 cells/mm^2^ at 3 months, and 1548 ± 401 cells/mm^2^ at 6 months postoperatively (40.7 ± 11.2% less than the preoperative value of the donor graft). In the control group, the donor corneal ECD decreased from 2707 ± 238 cells/mm^2^ preoperatively to 2021 ± 466 cells/mm^2^ at 1 month, 1837 ± 440 cells/mm^2^ at 3 months, and 1679 ± 419 cells/mm^2^ at 6 months postoperatively (38.2 ± 18.6% less than the preoperative value of the donor graft). There was no significant difference in ECD between the two groups at any pre- and postoperative points (Figure 2). The *P* values were 0.832 preoperatively, 0.136 at 1 month, 0.259 at 3 months, and 0.526 at 6 months.

### 3.5. Graft Unfolding Time

**Figure 3:**
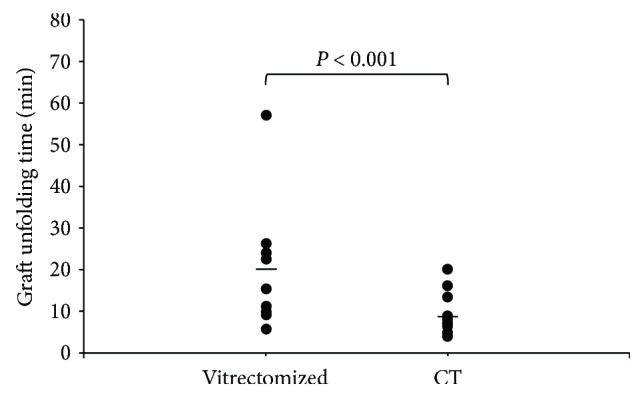
Comparison of graft unfolding time. Graft unfolding time was significantly longer in the vitrectomized group than in the control group (^∗^*P* < 0.001). The geometric mean graft unfolding time (indicated with bars) was 19.0 min in the vitrectomized group and 7.1 min in the control group. Vitrectomized: vitrectomized group; CI; confidence interval; CT; control group.

The geometric mean of the graft unfolding time was 19.0 min in the vitrectomized group (95% confidence interval (CI), 13.4–24.7) and 7.1 min in the control group (95% CI, 3.2–10.9). The graft unfolding time was significantly longer in the vitrectomized group than the control group (*P* < 0.001; Figure 3).

### 3.6. Complications after DMEK

None of the eyes showed intraoperative complications. Rebubbling for partial detachment was required in two eyes (25.0%) of the vitrectomized group and in four eyes (26.7%) of the control group; no significant difference between the two groups was observed (*P* = 0.554). CME was present in four eyes (50.0%) in the vitrectomized group and two eyes (13.3%) in the control group (*P* = 0.081). In all affected eyes, the CME resolved with topical bromfenac and sub-Tenon injection of triamcinolone acetonide. The mean BSCVA at 6 months after DMEK was 0.11 ± 0.12 in the eyes without CME and 0.037 ± 0.14 in the eyes with CME (*P* = 0.30).

### 3.7. BSCVA Prognosis-Related Factors

For BSCVA prognosis, we performed multiple regression analysis after stepwise variable selection. We used BSCVA at 6 months after DMEK as the response variable, and age, anterior chamber depth before DMEK, axial length, frequency of previous surgeries, graft unfolding time, and baseline BSCVA were examined as explanatory variables. The results are summarized in Table 3. After stepwise selection, age (estimated value = 0.006, *P* = 0.007) and frequency of previous surgeries (estimated value = 0.015, *P* < 0.001) were obtained as significant factors.

## 4. Discussion

The present investigation indicated that DMEK could be successfully performed in vitrectomized eyes with transscleral-sutured IOL, even though the graft unfolding times were significantly longer in the vitrectomized eyes. BSCVA was significantly improved in both groups, and postoperative CCT, ECD, and complication rates were comparable between the two groups.

In vitrectomized eyes, unfolding the graft can be challenging because the anterior chamber becomes shallow. Indeed, the geometric mean of the graft unfolding time was 19.0 min in the vitrectomized group, significantly longer than that of the control group (7.1 min; *P* < 0.001). This result is consistent with previous findings [23]. Yoeruek et al. reported 55% rebubbling rates after DMEK for postvitreous surgery, while 10% showed iatrogenic primary graft failure in the immediate postoperative period [15]. Weller et al. reported that graft detachments were observed in 45.8% of cases [14]. These previous findings indicate that longer manipulation to unfold the graft may cause more endothelial damage to the transplanted grafts. Fortunately, there was no primary graft failure, and the rebubbling rates were 22.2% in the vitrectomized group and 26.7% in the controls, with no group difference. Similarly, the ratio of ECD decrease at 6 months after DMEK was comparable between the two groups, with 40.7 ± 11.2% in the vitrectomized group and 38.2 ± 18.6% in the control group. These results indicate that careful preparation and manipulation during surgery might have contributed to successful DMEK even in the vitrectomized eyes.

The results suggest some important points for performing DMEK on the eyes that have undergone transscleral-sutured IOL implantation combined with PPV. First, an intact iris-IOL diaphragm is necessary. In such eyes, anterior segment reconstruction by suturing the iris could be necessary before DMEK. In the present investigation, we performed anterior segment reconstruction in four of the eight eyes in the vitrectomized group. Second, the position of the transscleral-sutured IOL is important. If the intraocular lens was sutured far posterior from the iris, the graft could be lost into the interspace between the iris and IOL or fall into the vitreous cavity [24]. Careful preoperative selection of the eyes and appropriate anterior segment reconstruction are thus necessary for successful DMEK.

Although the postoperative BSCVA significantly improved in both the vitrectomized and the control groups, it was also significantly lower in the vitrectomized group than in the control group. To clarify the underlying cause for this difference, we performed multiple regression analysis after stepwise variable selection for BSCVA prognosis, and the results showed that the patients' age and the frequency of the previous surgeries were highly related to the postoperative BSCVA. Similar findings that the visual acuity of younger patients tended to improve have also been reported after other ocular surgeries, such as cataract surgery and vitrectomy for macular hole [25–27]. Several factors, including unrecognized or subclinical comorbidity, age-related changes in macular function, and the tendency to perceive functional impairment irrespective of vision in elderly people, could have contributed to these differences [28].

Another important factor affecting postoperative BSCVA was the frequency of previous surgeries. Postoperative BSCVA has been reported to be worse for previous repeated intraocular surgeries including penetrating keratoplasty, Boston keratoplasty, and PPV [29–31]. Repeated surgeries may cause persistent inflammation, elevated intraocular pressure, and/or insufficient ocular circulation, resulting in deteriorated retinal function. Moreover, precisely centering the IOL can be difficult for the eyes with transscleral-sutured IOL, also contributing to BSCVA impairment. However, this aspect was not evaluated in the present study. The worse BSCVA in the vitrectomized group compared with the control group may thus be caused by various factors.

Flanary et al. reported that the incidence of CME was 8.0% (7 among 88 eyes) after staged DMEK that was performed within 6 months after cataract surgery and 7.1% (6 among 85 eyes) in solitary DMEK performed more than 6 months after cataract surgery [32]. According to a cohort study by Heinzelmann et al., 13% of the eyes developed a single episode of CME during the follow-up time after DMEK [33]. In the current study, the incidence of development of CME was similar to that of previous reports in the control group (13.3%), but much higher in the vitrectomized group (50.0%), although there was no significant difference between the two groups. Furthermore, CME occurrence was not significantly correlated with postoperative BSCVA (0.110 versus 0.037, resp.; *P* = 0.300). We speculate that this is probably because we applied topical bromfenac and sub-Tenon injection of triamcinolone acetonide immediately upon the detection of CME postoperatively. However, further studies are still needed to ascertain the influence of CME on the final visual outcome after DMEK.

Our findings indicate that we could obtain comparable outcomes with respect to the mean ECD and complication rates using DMEK for typical pseudophakic nonvitrectomized eyes. Moreover, DMEK could produce excellent visual outcomes and low rejection rates. Even in complex cases such as vitrectomized eyes, we observed impressive visual recovery after DMEK. In fact, the BSCVA in one case improved from 20/200 to 20/50, despite the fact that the patient had been limited to 20/100 after previous DSAEK. Caution should be exercised in the selection of the candidates, including presurgical preparation such as creating an intact iris-IOL diaphragm. Careful postoperative evaluation of the occurrence of CME and its immediate treatment may also contribute to improvement of the surgical outcomes.

## 5. Conclusion

In conclusion, DMEK can improve visual function in the eyes that underwent previous PPV and transscleral-sutured IOL implantation.

## Figures and Tables

**Table 1:** Patient characteristics before surgery.

	Vitrectomized group	Control group	*P* ^∗^
Number of eyes	8	15	
Sex (male/female)	5/3	3/12	0.051^∗^
Age	72.8 ± 10.5	74.1 ± 5.1	0.906^†^
Eye (R/L)	3/5	7/8	0.632^∗^
BSCVA (LogMAR)	1.15 ± 0.60	0.98 ± 0.52	0.795^†^
CCT (*μ*m)	765 ± 63	722 ± 88	0.194^†^
Frequency of previous surgeries	3.11 ± 0.78	1	<**0.001**

BSCVA: best spectacle-corrected visual acuity; CCT: central corneal thickness; L: left; LogMAR: logarithm of the minimal angle of resolution; R: right. ^∗^*χ*^2^ test (comparison between two groups); ^†^unpaired *t*-test.

**Table 2:** Profiles of the enrolled patients (vitrectomized group).

Case	Sex	Age	OD/OS	Etiology for PPV	Previous surgeries	Preop BSCVA	Preop CCT (*μ*m)	Treatment before DMEK
1	F	79	OD	PEX	PEA, PPV + IOLs	20/2000	793	
2	M	79	OS	PEX	ICCE, PPV + IOLs	20/1000	724	
3	F	74	OS	PEX	ICCE + PPV + IOLs, DSAEK	20/200	836	ASR
4	F	52	OS	Extended CL wearing	Cataract aspiration, PPV + IOLs	20/50	672	
5	F	64	OS	Dropped IOL	PPV + IOLs	20/2000	734	ASR
6	F	56	OS	RRD	PPV + SOi, SOr + IOLs	20/50	757	
7	M	79	OD	RRD	PPV + SOi, SOr + IOLs	20/500	939	ASR
8	M	74	OD	Endophthalmitis	PPV + IOLr + SOi, SOr + IOLs	20/100	658	ASR

ASR: anterior segment reconstruction; BSCVA: best spectacle-corrected visual acuity; CCT: central corneal thickness; DMEK: Descemet membrane endothelial keratoplasty; ICCE: intracapsular cataract extraction; IOLr: removal of intraocular lens; IOLs: transscleral-sutured posterior chamber intraocular lens implantation; OD: right eye; OS: left eye; PEA: phacoemulsification and aspiration; PEX: pseudoexfoliation syndrome; PPV: pars plana vitrectomy; Preop: preoperative; RRD: rhegmatogenous retinal detachment; SOi: silicone oil injection; SOr: silicon oil extraction.

**Table 3:** Multiple regression analysis for correlates of postoperative best spectacle-corrected visual acuity (BSCVA).

Predictor	Estimated value	SE	*P* value
Age	0.00061	0.0021	**0.009**
AXL	Unselected		0.95
ACD	Unselected		0.26
Frequency of previous surgeries	0.11	0.016	**<0.001**
Unfolding time	Unselected		0.95
CME	Unselected		0.45
Preoperative BSCVA	Unselected		0.68

SE: standard error; AXL: axial length; ACD: anterior chamber depth; CME: cystoid macular edema. Multivariate analysis was constructed after stepwise variable selection (BIC, forward method).
